# Microstate Detection in Naturalistic Electroencephalography Data: A Systematic Comparison of Topographical Clustering Strategies on an Emotional Database

**DOI:** 10.3389/fnins.2022.812624

**Published:** 2022-02-14

**Authors:** Wanrou Hu, Zhiguo Zhang, Li Zhang, Gan Huang, Linling Li, Zhen Liang

**Affiliations:** ^1^School of Biomedical Engineering, Health Science Center, Shenzhen University, Shenzhen, China; ^2^Guangdong Provincial Key Laboratory of Biomedical Measurements and Ultrasound Imaging, Shenzhen, China; ^3^Peng Cheng Laboratory, Shenzhen, China; ^4^Marshall Laboratory of Biomedical Engineering, Shenzhen, China

**Keywords:** EEG, microstate detection, naturalistic task, topographical clustering, bottom-up, top-down, performance evaluation

## Abstract

Electroencephalography (EEG) microstate analysis is a powerful tool to study the spatial and temporal dynamics of human brain activity, through analyzing the quasi-stable states in EEG signals. However, current studies mainly focus on rest-state EEG recordings, microstate analysis for the recording of EEG signals during naturalistic tasks is limited. It remains an open question whether current topographical clustering strategies for rest-state microstate analysis could be directly applied to task-state EEG data under the natural and dynamic conditions and whether stable and reliable results could still be achieved. It is necessary to answer the question and explore whether the topographical clustering strategies would affect the performance of microstate detection in task-state EEG microstate analysis. If it exists differences in microstate detection performance when different topographical clustering strategies are adopted, then we want to know how the alternations of the topographical clustering strategies are associated with the naturalistic task. To answer these questions, we work on a public emotion database using naturalistic and dynamic music videos as the stimulation to evaluate the effects of different topographical clustering strategies for task-state EEG microstate analysis. The performance results are systematically examined and compared in terms of microstate quality, task efficacy, and computational efficiency, and the impact of topographical clustering strategies on microstate analysis for naturalistic task data is discussed. The results reveal that a single-trial-based bottom-up topographical clustering strategy (bottom-up) achieves comparable results with the task-driven-based top-down topographical clustering (top-down). It suggests that, when task information is unknown, the single-trial-based topographical clustering could be a good choice for microstate analysis and neural activity study on naturalistic EEG data.

## Introduction

Electroencephalography (EEG) is an efficient and reliable neuroimaging technique for tracking the dynamic changes of physiological brain states. In the field of joint spatial-temporal EEG study, EEG microstate analysis is recommended as a powerful method for brain mechanism investigation through an inspection of the spatial changes of EEG potential distribution along the time (Khanna et al., 2015; Michel and Koenig, 2018). Different from traditional EEG analysis methods, EEG microstate analysis highly relied on a topographical clustering strategy to identify the ***microstates*** from spontaneous EEG activity, as every data time point would be assigned to one microstate class based on the spatial similarity to the identified microstate templates (Murray et al., 2008; Brunet et al., 2011; Michel and Koenig, 2018). Notably, EEG microstates characterize the quasi-stable potential distributions of whole-brain EEG activities, which are also termed **microstate templates** (Khanna et al., 2015; Michel and Koenig, 2018). Through analyzing the dynamic temporal variations in microstate templates, the **microstate patterns** are obtained and the momentary fluctuations of spatial-temporal EEG activities during naturalistic task manipulation are quantitively estimated. Therefore, the successful detection of reliable microstate templates is of great significance in characterizing the temporal alternations in EEG microstate activity patterns for characterizing task-related functional brain dynamics.

Current EEG microstate analysis mainly works on rest-state EEG recordings, based on a simple and fixed topographical clustering strategy. For example, Xu et al. (2020) conducted a trial-subject-based clustering analysis on rest-state sleeping EEG data for estimating the functional association between EEG microstates and fMRI networks. In their case, a trial-based topographical clustering was first conducted on every single trial of EEG data to identify subject-specific microstate candidates. Then, in a cross-subject manner, another clustering analysis was performed to detect EEG microstate templates from all topographical candidates identified by the trial-based clustering. A similar topographical clustering strategy was also used in Brodbeck et al.’s (2012) work for sleep staging based on the rest-state EEG data. Such a trial-subject-based clustering strategy is frequently used in rest-state EEG microstate analysis. On the other hand, to characterize the brain state differences between patients with mild cognitive impairment and healthy controls, Musaeus et al. (2019) introduced a subject-independent clustering strategy to identify common microstate templates from these two groups of EEG recordings. They mixed all the resting EEG data from patients and healthy people together for one-step microstate clustering in a cross-subject manner and obtained a set of common EEG microstate templates.

Besides the rest-state EEG studies, researchers also try to introduce EEG microstate analysis to task-state EEG studies for inspecting the dynamic brain activity during task manipulation. For example, Han et al. (2020) adopted EEG microstate analysis into the neural mechanism of facial attractiveness judgment and found that high attractive faces would significantly activate microstate responses of MS3. From a dynamic perspective into brain responses, task-state EEG microstate analysis would provide us with an informative and novel detection of neural processing mechanisms. Current studies show a possibility to directly apply a similar clustering strategy (e.g., trial-subject-based topographical clustering) from rest-state EEG study to task-state EEG microstate analysis. For example, in Gui et al.’s (2020) study, the microstate candidates were first extracted across all the trials for each subject (cross-trial-level) and then fused across all the subjects for microstate template detection (cross-subject-level). Different from rest-state EEG studies, task manipulation would dramatically influence the mental and cognitive states and lead to specific changes in spontaneous brain activities (Milz et al., 2016; Seitzman et al., 2017; D’Croz-Baron et al., 2021). Therefore, on the basis of the existing clustering strategies used in resting EEG microstate analysis, the different input orders of EEG data and clustering arrangement were also considered. For example, in Milz et al. (2016)’s work, to explore the brain activity differences under three thinking states (object recognition, spatial identification, verbalization), they conducted a complex microstate detection analysis with three steps of topographical clustering. Firstly, for each subject, subject-specific microstate candidates were extracted by grouping all trials of EEG data under the same thinking tasks. Secondly, for each modality of thinking tasks, task-specific microstate candidates were identified by conducting the cluster analysis across all the subject-specific microstate candidates. Thirdly, the final microstate templates were identified by clustering task-specific microstate candidates together. When conducting naturalistic tasks, our human brain is dynamic alternating in response to the ever-changing stimulation. Therefore, task-state brain activities are complex with rapid temporal variations and great individual diversity. It is a critical problem in task-state EEG microstate analysis that how to identify reliable microstate templates by a suitable topographical clustering strategy for properly describing the high EEG variability and complexity during task manipulation. A good EEG microstate detection with high-quality brain state representation would directly benefit the following time-series data presentation and analysis. Therefore, the performance of topographical clustering strategies in microstate template detection is a critical topic in naturalistic task-state EEG microstate analysis.

In the process of microstate detection, EEG microstates are identified as the potential topographies at stable centroids of topographical clustering analysis (Pascual-Marqui et al., 1995; Skrandies, 2007). Here, the adopted topographical clustering strategies could have several options (Khanna et al., 2014), e.g., data order, clustering arrangement, and whether task information is used as guidance. However, a few current studies have considered the impact of topographical clustering strategies on microstate detection performance. Systematic analysis is needed to compare the microstate detection performance of different clustering strategies, which benefit the exploration of an optimal, reliable, and efficient microstate detection method for naturalistic task-state EEG microstate analysis. In this work, we introduce a total of 5 types of topographical clustering strategies in task-state EEG microstate detection and compare the performance differences on the same EEG database collected under a naturalistic paradigm. The effect of clustering sequences with different date grouping order and clustering arrangement is explored, and the role of task-prior knowledge in microstate analysis is discussed. Here, the task data are grouped in terms of trials, subjects, or random clusters, and the clustering sequences, e.g., first trial then subject (trial-subject-sequence-based), first subject then trial (subject-trial-sequence-based), only trial (single-trial-based), and random clusters (random-grouping-based). For the characterization of neural patterns correlated to naturalistic simulation, we estimate the performance differences when task-prior knowledge is adopted (***top-down***) or not adopted (***bottom-up***) in the topographical clustering process. An ideal data-driven bottom-up topographical clustering strategy is expected to achieve a close result to the knowledge-guided top-down topographical clustering strategy. To well evaluate microstate detection results and conduct a careful comparison among different topographical clustering strategies, we also introduce a full performance evaluation protocol to verify the detected microstates from three perspectives: microstate quality, task efficacy, and computational efficiency. In all, this work mainly focuses on the microstate detection performance in naturalistic task-state EEG microstate analysis. And our main contributions include: (1) we estimate and compare the topographical clustering strategies in microstate detection from naturalistic task-state EEG data; (2) we evaluate the prior knowledge impact on microstate clustering; (3) we present a systematic evaluation protocol for microstate detection performance validation. Our work offers supportive guidelines in choosing a suitable and reliable topographical clustering strategy in task-state EEG microstate detection and will benefit the development of EEG microstate analysis to characterize the neural dynamic activities under naturalistic paradigms.

## Materials and Methods

### Electroencephalography Database and Data Preprocessing

Electroencephalography signals cover informative electrophysiological evidence that is beneficial for effective and reliable affective state estimation (Alarcão and Fonseca, 2019). The public emotional database constructed by Koelstra et al. (2012) (A Database for Emotion Analysis using Physiological Signals, DEAP) is widely used as a benchmark for EEG-based video-evoking emotion study. In this database, 40 1-min music videos with outstanding emotion-evoking performance were carefully selected and utilized as emotion-evoking materials, and 32 healthy subjects were recruited for spontaneous, dynamic, and naturalistic emotion induction experiments. For each trial, it consisted of a 5-s baseline with a fixation cross displaying in the monitor center and a 60-s video-viewing in which one video was randomly played for emotion-evoking. Subsequently, a self-assessment of different emotional dimensions, e.g., valence and arousal, was conducted for subjective feedback based on the evoked emotion states during video-viewing. Simultaneously, 32-electrode EEG signals were recorded by the Biosemi ActiveTwo system at a sampling rate of 512 Hz. In this paper, the emotional EEG data from the DEAP database are used to estimate the performance of different microstate detection methods in the task-state EEG analysis.

As EEG data are susceptibly impeded by unrelated artifacts (caused by ocular movement and blinking, muscular activity, etc.), EEG processing is first conducted for noise removal to enhance the data quality. Based on the collected raw EEG data, a standard EEG preprocessing procedure is conducted as below. **(1) Filtering**: a bandpass filter between 1 and 45 Hz is applied for useful information selection, and a notch filter at the frequency of 50 Hz is utilized for line power artifact removal. **(2) Noisy channel exclusion**: the noisy channels are excluded and interpolated by the neighboring three channels. After interpolation, the noisy channels are replaced by the average amplitude of their neighboring channels. **(3) Re-reference**: a common average re-reference calculation is conducted for random noise removal. Afterward, the EEG data are adjusted to a zero-mean distribution. **(4) Independent component analysis (ICA)**: the noisy components after ICA decomposition are manually rejected, and then clean EEG signals are reconstructed from the artifact-clean components. **(5) Segmentation**: only the EEG data recorded at the video-watching (60 s) are detected for task-state EEG analysis.

After the data preprocessing, a total of 1280 trials (32 subjects × 40 trials) of artifact-clean task-state EEG recordings are obtained for following EEG microstate analysis. In this study, a Microstate EEGLAB Toolbox (Poulsen et al., 2018) is adopted for task-state EEG microstate analysis.

### Standard Electroencephalography Microstate Analysis

Electroencephalography microstate analysis is a powerful method for dynamic brain activity analysis (Van De Ville et al., 2010). The main idea of EEG microstate analysis is to detect a few quasi-stable EEG potential topographies for characterizing the dynamic spatial-temporal changes of original EEG signals (Khanna et al., 2015; Michel and Koenig, 2018). These identified EEG topographies are termed EEG microstates with quasi-stable potential distributions around 80–120 ms (Lehmann and Skrandies, 1980). The momentary fluctuation of the whole-brain EEG activity can be presented as the dynamic transition among EEG microstates with millisecond temporal resolution. Generally, a standard procedure of EEG microstate analysis includes four stages. **(1) Candidate topography extraction**. The preprocessed EEG data are utilized as the input for EEG microstate analysis. To search for quasi-stable EEG topographies, potential topographies with a high signal-to-noise ratio are first extracted. Here, global field power (GFP) of each EEG sample point is measured as a reference-independent quantification of the whole-brain potential power, given as


(1) $$\text{GFP}=\sqrt{\frac{1}{N}\sum_{i=1}^{N}{\left(V_{i}\left(t\right)-\bar{V\left(t\right)}\right)}^{2}},$$

where *V*_*i*_(*t*) refers to the voltage of *i*th electrode at the time point *t*; $\bar{V\left(t\right)}$ is the average voltage across all the electrodes over the whole brain at the given time *t*, calculated as


(2) $$\bar{V\left(t\right)}=\sqrt{\frac{1}{N}\sum_{i=1}^{N}V_{i}\left(t\right)}.$$

here, *N* is the total number of EEG electrodes. Previous studies have fully explored that topographies at the GFP peak (local maxima) correspond to a high signal-to-noise ratio, which are well-reasoned and stable candidates for representing spontaneous brain activities (Lehmann and Skrandies, 1980; Lehmann et al., 1987; Zanesco, 2020). All the potential topographies at local maximal points of the GFP are detected in a data-driven manner and extracted from each EEG recording as the **candidate templates** for further clustering analysis. **(2) EEG microstate detection**. Based on the extracted candidate topographies obtained in Stage 1, a series of microstates are identified in a data-driven manner. A topographical clustering is conducted on all the extracted candidate topographies and the cluster centroids with the highest spatial similarity during iterating are calculated as the ***microstates***. In the implementation, the polarity of candidate topographies is ignored, as the spontaneous EEG topographies with inverted polarity share a synchronized and similar activation pattern of neuronal ensembles (Lehmann and Koenig, 1997; Mishra et al., 2020). According to the studies presented in Khanna et al. (2014), Von Wegner et al. (2018), it was found that k-Means clustering and hierarchical clustering (such as Atomize and Agglomerate Hierarchical Clustering and Topographic Atomize and Agglomerate Hierarchical Clustering) yielded equal performance in microstate detection on spontaneous EEG activities. The reason is that these two clustering algorithms follow a similar calculation criterion of ignoring potential polarity and detecting cluster centroids based on topographical configuration. Thus, in this work, to have a fair comparison across different topographical clustering strategies, a modified k-means clustering is consistently used for microstate detection, which is the most commonly used clustering method in EEG microstate analysis (Pascual-Marqui et al., 1995). **(3) EEG microstate segmentation**. A spatial correlation is measured between every EEG microstate and momentary potential topography, and every EEG time point is then assigned to one EEG microstate that yields the highest spatial similarity. Therefore, the original EEG data are represented by a series of EEG microstates that cover rich information on spatial-temporal dynamics of brain activities under emotion induction. **(4) Microstate feature extraction**. For quantifying the dynamic characteristics of naturalistic task-state EEG data under video-viewing emotion induction, EEG microstate features (e.g., duration, coverage, occurrence, and transition probability) are extracted, and the feature distributions under different emotional states are studied.

Among the above four stages in the standard EEG microstate analysis pipeline, the performance of EEG microstate detection in stage (2) is easily influenced by the adopted topographical clustering strategy, whose results will further impact the reliability of the final identified microstates. In current existing task-state EEG studies, a systematical investigation of the impact of the topographical clustering strategies in the EEG microstate detection is still lacking. Therefore, in this work, following the same parameter setting in candidate topography extraction, EEG microstate segmentation, and microstate feature extraction, we mainly examine and discuss the effect of topographical clustering strategies in EEG microstate detection with different data grouping order, clustering arrangement, and whether using task information for guidance.

### Electroencephalography Microstate Detection With Different Topographical Clustering Strategies

**FIGURE 1:**
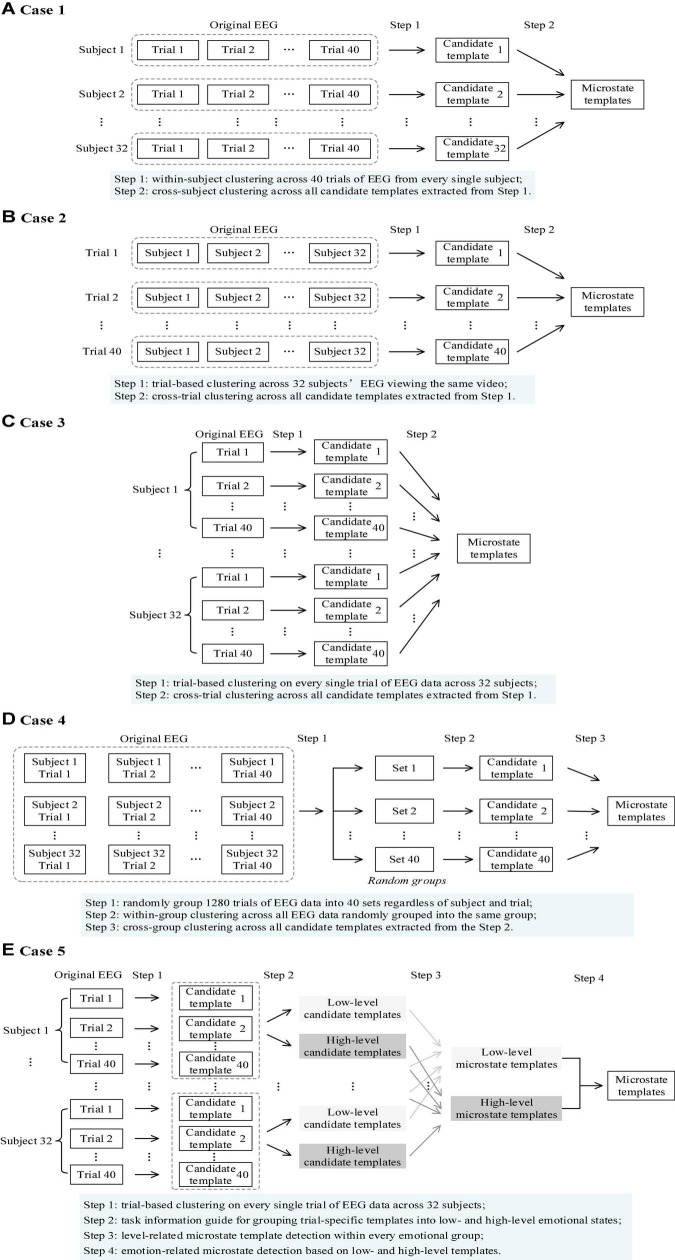
A schematic overview of five topographical clustering strategies for task-state microstate detection. **(A)** A trial-subject-sequence-based bottom-up topographical clustering strategy (Case 1, Section “A Trial-Subject-Sequence-Based Bottom-Up Topographical Clustering”). **(B)** A subject-trial-sequence-based bottom-up topographical clustering strategy (Case 2, Section “A Subject-Trial-Sequence-Based Bottom-Up Topographical Clustering”). **(C)** A single-trial-based bottom-up topographical clustering strategy (Case 3, Section “A Single-Trial-Based Bottom-Up Topographical Clustering”). **(D)** A random-grouping-based bottom-up topographical clustering (Case 4, Section “A Random-Grouping-Based Bottom-Up Topographical Clustering”). **(E)** A task-driven-based top-down topographical clustering (Case 5, Section “A Task-Driven-Based Top-Down Topographical Clustering”).

In the following, four task-free topographical clustering strategies (bottom-up-based data-driven approaches, referring to Case 1, 2, 3, 4 below) and one task-guided topographical clustering strategy (top-down-based prior-knowledge guided, referring to Case 5 below) are presented, examined, and compared with full details (Figure 1).

#### A Trial-Subject-Sequence-Based Bottom-Up Topographical Clustering (Case 1)

In the trial-subject-sequence-based bottom-up topographical clustering strategy (named as Case 1 below), a similar clustering strategy commonly used in resting-state EEG microstate analysis is adopted. The main idea of this clustering strategy is: subject-specific candidate templates are first extracted across trials of EEG data and then fused for subject-generalized (or subject-independent) EEG microstates identification (Figure 1A). Here, two steps of topographical clustering are involved. **Step 1:** for each subject, a topographical clustering is conducted on 40 trials of EEG data. The first-step clustering is conducted in a within-subject manner to characterize subject-specific electrophysiological responses during different emotional video watching (different mental states). For every single subject, potential topographies are extracted from 40 trials to form a candidate template (subject-specific candidate templates). **Step 2:** a topographical clustering is conducted on all the obtained subject-specific candidate templates. The subject-generalized microstate templates are obtained as the final microstates.

#### A Subject-Trial-Sequence-Based Bottom-Up Topographical Clustering (Case 2)

In the subject-trial-sequence-based bottom-up topographical clustering strategy (named as Case 2 below), the task-initiated electrophysiological brain activities are considered to learn about the neural mechanism of dynamic information processing under specific tasks. The main idea of this clustering strategy is: the candidate templates are detected for each task (in our case, each video corresponds to a task) based on the collected 32 subjects’ EEG data under the same video stimulation and the common patterns of video-initiated spontaneous brain activities are captured (Figure 1B). Here, two steps of clustering with data grouping order of across subjects and then across trials are involved. **Step 1**: a cross-subject topographical clustering is conducted on all the subjects’ EEG data triggered by the same video clip. As 40 videos are randomly represented to each subject in the experimental data collection, we first reorder all the EEG recordings according to the video order. Then, for each video, EEG recordings from 32 subjects are used for candidate template detection, and video-specific microstate candidate templates are identified. **Step 2**: a topographical clustering is conducted on all the obtained candidate templates from a total of 40 videos. An optimal set of quasi-stable microstate templates is extracted and the video-generalized (or video-independent) EEG microstates are obtained.

#### A Single-Trial-Based Bottom-Up Topographical Clustering (Case 3)

In the previous two cases (Case 1 and 2), the topographical clustering at Step 1 is conducted on subject-specific multiple videos (Case 1) or video-specific multiple subjects (Case 2). It could be also possible to conduct candidate template detection on a single trial of EEG recordings for trial-specific candidate template extraction in the first-step clustering. Then, all the candidate templates are further grouped in the second-step clustering for final EEG microstate detection. Following this clustering strategy, the detailed process of the single-trial-based bottom-up topographical clustering strategy (named as Case 3 below) is described here (Figure 1C). **Step 1:** a topographical clustering is conducted on every single trial of EEG recordings. The first-step clustering is conducted on trial-based EEG data, and a set of trial-specific microstate candidate templates are identified. **Step 2**: a topographical clustering is conducted on all the obtained trial-specific candidate templates obtained in Step 1. We gather all the topographies from first-step clustering together and submit them to another clustering analysis for final EEG microstate template detection. Therefore, the second-step clustering of Case 3 is conducted at a trial-independent level.

#### A Random-Grouping-Based Bottom-Up Topographical Clustering (Case 4)

The previous three cases are all order-orientated clustering strategies for EEG microstate detection, where an arrangement of input orders is designed at subject-level (Case 1), video-level (Case 2), and trial-level (Case 3). To verify the ordering impacts on the microstate detection performance, a random-grouping-based bottom-up topographical clustering strategy (named as Case 4 below) is considered, where all the trials are randomly grouped into different sets for two-step topographical clusterings (Figure 1D). **Step 1**: all trials of EEG recordings are randomly grouped. For the DEAP database, a total of 1280 EEG recordings (32 subjects × 40 trials) are randomly divided into 40 groups without consideration of the information about trials and subjects. In other words, for each group, the included EEG recordings are from different trials and subjects. **Step 2**: a topographic clustering is conducted on each group. In total, 40 sets of group-specific candidate templates are obtained. **Step 3**: a topographical clustering is conducted on all the group-specific candidate templates obtained in Step 2. A final set of microstates are obtained across 40 random groups.

#### A Task-Driven-Based Top-Down Topographical Clustering (Case 5)

Besides the above-mentioned four data-driven-based topographical clustering strategies, we also evaluate the topographical clustering performance under task information guidance. As emotion is a subjective experience, the self-assessment offers an effective way for evoking emotion estimation and is generally accepted as the ground truth (labels) for emotion understanding in individuals (Bradley and Lang, 1994; Alarcão and Fonseca, 2019). For the DEAP database, the task information guidance is the reported subjective feedbacks after video-watching. In the task-driven-based top-down topographical clustering strategy (named as Case 5 below), the subjective feedbacks (emotion labels) of valence and arousal emotional dimensions are adopted for microstate detection, and the corresponding valence/arousal based microstates are identified, respectively (Figure 1E). Here, four steps are involved. **Step 1**: a trial-based topographical clustering is conducted. Similar to Step 1 in Case 3, the trial-specific candidate templates are detected. **Step 2**: a label-guided data grouping is conducted for each subject. The subjective labels (9-point self-assessment ratings) are discretized into binary levels (low-level and high-level). For each subject, a subject-specific adaptive threshold calculation (Yin et al., 2017) is adopted to divide the self-assessment ratings in valence and arousal dimensions into two clusters through a k-means algorithm. The midpoint of two cluster centroids is used as the threshold for binary grouping. **Step 3**: a topographical clustering is conducted on each emotional level for each subject. For each subject, the corresponding low-level and high-level candidate templates are identified for valence and arousal, respectively. In other words, two pairs of candidate templates are obtained for each subject, which reflects the synchronized oscillatory of whole-brain EEG activities under different emotion states. **Step 4**: a topographical clustering is conducted on the obtained low- and high-level candidate templates. Note that valence and arousal are two independent emotional dimensions, thus we explore the neural mechanisms underlying these two emotional dimensions separately. In other words, the valence-based and arousal-based microstates are, respectively, detected based on the corresponding low- and high-level candidate templates.

### Performance Evaluation Protocol

To systematically quantify the microstate detection performance on task-state EEG data, we explore different topographical clustering strategies (from Case 1 to Case 5) and evaluate the corresponding identified ***microstates*** from three perspectives: **microstate quality**, **task efficacy**, and **computational efficiency**. Here, microstate quality mainly focuses on the topographical characteristics of EEG microstates, including the measurements of the electric potential distribution (spatially) and the global fitness in EEG microstate segmentation (temporally). Task efficacy is an evaluation of whether the identified microstates can reflect the changes in mental states under task manipulation. Computational efficiency measures the total cost time on topographical clustering for EEG microstate detection.

#### Microstate Quality

Electroencephalography microstate topographies reflect the global patterns of momentary potential distribution over whole-brain scalp EEG (Lehmann et al., 1987; Michel and Koenig, 2018), which determine the data quality and spatial-temporal property of the final EEG microstate sequences. Here, microstate quality is evaluated in terms of spatial correlation and global explained variance measurements.

##### Spatial Correlation

The spatial similarities among EEG microstate topographies can be quantified by a correlation-based measurement. The global map dissimilarity (GMD) is a global measurement of topographical differences between two potential maps, calculated as


$$GMD=\sqrt{\frac{1}{N}\sum_{i=1}^{N}{\left\{\frac{p_{i}-\bar{p}}{\sqrt{\frac{1}{N}\sum_{i=1}^{N}{\left(p_{i}-\bar{p}\right)}^{2}}}-\frac{q_{i}-\bar{q}}{\sqrt{\frac{1}{N}\sum_{i=1}^{N}{\left(q_{i}-\bar{q}\right)}^{2}}}\right\}}^{2}},$$

where *p_i_* and *q_i_* refer to the voltage of topography map *p* and *q* at *i*th electrode, and $\bar{p}$ and $\bar{q}$ are the corresponding average voltages across whole-brain electrodes of map *p* and *q*. *N* is the electrode number of EEG recordings. The spatial correlation between potential topographies is given as


(4) $$R=1-\frac{GMD^{2}}{2}.$$

Numerically, it is equivalent to Pearson’s correlation coefficient between potentials of two EEG topographies (Brunet et al., 2011). Spatial correlation is a strength-independent evaluation of topographical similarity (Murray et al., 2008), and it has been commonly used as a criterion for EEG microstate segmentation by measuring the spatial similarity between original topographies at each sampling point and EEG microstate templates (Koenig et al., 1999; Lehmann et al., 2005). To quantify the quality of the identified EEG microstates under different topographical clustering strategies, the spatial correlation is first evaluated with the detected task-state microstate templates from a well-known public set (four microstates with canonical potential configuration are clustered from 61 healthy subjects under four modalities of thinking states). Then, the spatial correlation is also examined with the detected microstate templates under the guidance of task information (Case 5) to check whether the identified microstates in a bottom-up manner could share a similar neuronal activity with the emotion-evoked brain states detected in a top-down manner (Yuan et al., 2012; Vellante et al., 2020).

##### Global Explained Variance

The global explained variance (GEV) is another important evaluation index that reflects the retained information of the original EEG data in the identified EEG microstates. GEV measures the overall spatial correlation between the EEG maps at all time points and each microstate (Koenig et al., 2002; Mishra et al., 2020), given as


(5) $$GEV=\frac{\sum_{t=1}^{L}{\left(Corr\left(V\left(t\right),V_{c}\left(t\right)\right)\cdot GFP\left(t\right)\right)}^{2}}{\sum_{t=1}^{L}GFP^{2}\left(t\right)},$$

where *V*(*t*) stands for EEG map at the given time *t*, and *V*_*c*_(*t*) denotes the microstate that is assigned to this time point. Corr(⋅) represents the correlation calculation of spatial similarity between two given topographies. GFP(*t*) is the global field power at time point *t*. *L* is the total number of time points in EEG recordings.

#### Task Efficacy

Task efficacy is to evaluate whether the identified microstates could well reflect the corresponding task information involved in the task-state EEG recordings. Among the presented five different types of EEG microstate detection methods with different topographical clustering strategies, only the task-driven-based top-down topographical clustering (Case 5) utilize the task information involved in the task-state EEG recordings; the other four cases (Case 1–4) are pure data-driven based bottom-up approaches, without any knowledge guidance about the task. In the other words, we could consider the top-down method (Case 5) should perform superior in task efficacy compared to the bottom-up methods. An evaluation of task efficacy could become a question to measure the task representation differences between bottom-up and top-down methods.

Based on the microstates, an EEG microstate time series representation is formed for each trial of task-state EEG recordings, which cover rich information about dynamic changes of neurophysiological states during the task. Microstate features in terms in terms of duration, coverage, occurrence, and transition probability are characterized for task efficacy evaluation. Take the emotion task as an example. The task efficacy is evaluated to answer the three questions below. (1) Whether the characterized microstate features could reflect the emotion levels? (2) Which microstate features characterized by the task-driven-based top-down topographical clustering method are highly related to the emotion changes? (3) Which case of bottom-up topographical clustering methods share similar feature patterns in the reflection of emotion changes compared to the top-down method’s results? Here, in terms of each microstate feature characterized by each case of EEG microstate detection method, the pattern differences between low- and high-level emotion states are examined in valence and arousal dimensions, respectively. Based on a Lilliefors test for normality distribution estimation, we choose an independent *t*-test to examine the feature data that follow a normal distribution and adopt a Wilcoxon Rank Sum test for the data that do not follow a normal distribution. After correction for multiple comparisons using false discovery rate (FDR), the *p*-values results of the two-side test present a statistical quantification about whether the pattern differences between two different emotion levels are large enough to satisfy the requirement of statistical significance. In other words, the microstate features that could reflect emotion changes should be statistically significant in the two-side test.

#### Computational Efficiency

**FIGURE 2:**
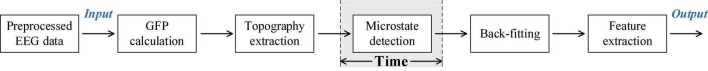
A schematic display of time monitoring on the computational efficiency of microstate detection.

We also measure the computational time of each topographical clustering strategy. This evaluation index could be used as supportive information for future EEG microstate detection method selection in practical and clinical applications. As shown in Figure 2, the computation efficiency is the time cost calculation of the entire microstate detection part, in which different topographical clustering strategies could be used.

## Results

All the five EEG microstate detection methods with different clustering strategies are evaluated on the same EEG recordings under a naturalistic paradigm (DEAP database; 32 subjects × 40 trials = 1280 trials). The final detected microstates are verified in terms of microstate quality, task efficacy, and computation efficiency.

### Microstates

**FIGURE 3:**
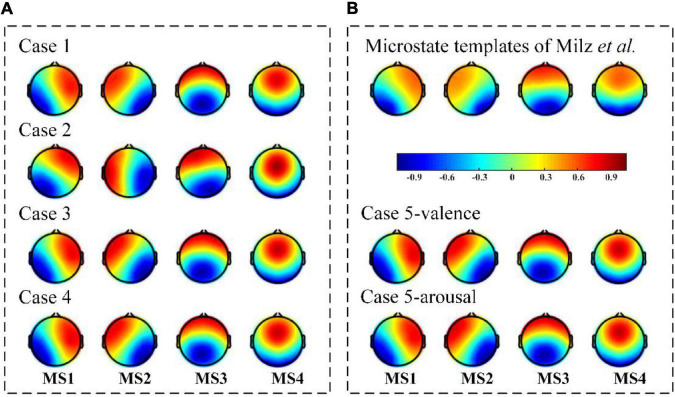
The final detected microstates with different topographical clustering methods. **(A)** The identified microstates by Case 1, Case 2, Case 3, and Case 4. All are bottom-up-based data-driven approaches. **(B)** The identified microstates by Milz et al. (2016) and Case 5. Milz et al.’s (2016) results could be considered as standards in the existing EEG microstate studies, in which the underlying neural mechanisms have been verified with functional magnetic resonance imaging studies. Case 5’s results could be more capable of reflecting the specific brain states under tasks, as the task information is used as a guide in the detection procedure (top-down-based task information-guided approach). A good microstate detection performance should share a similar topography distribution property with the standard templates and the task information-guided templates.

The final detected microstates using different topographical clustering strategies are presented in Figure 3. Note here Case 1 to Case 4 are bottom-up-based data-driven approaches and Case 5 is a top-down-based task information-guided approach. The results show that the identified microstates share a similar potential distribution with the canonical configuration in the previous study (Pascual-Marqui et al., 1995; Lehmann and Koenig, 1997; Milz et al., 2016; Michel and Koenig, 2018). According to the potential distributions given in Milz et al.’s (2016) study, the microstates are labeled as MS1 (right frontal-left posterior), MS2 (left frontal-right posterior), MS3 (midline frontal-to-occipital), and MS4 (frontocentral to occipital orientations). It is also found a left-right symmetry microstate is identified in Case 2 (the second map in row 2 of Figure 3A), which is topographically different from the canonical EEG microstates in the literature. Besides the visual inspection, we also conduct the quantitative analysis for performance evaluation and present the results below.

### Microstate Quality

The scalp potential distribution of EEG microstate templates reflects the momentary electrophysiological state of the brain and is coordinated with the whole-brain neuronal activities. In the evaluation of microstate quality, we mainly focus on the topographical stability (spatial property) of the identified microstate templates.

#### Spatial Correlation

**FIGURE 4:**
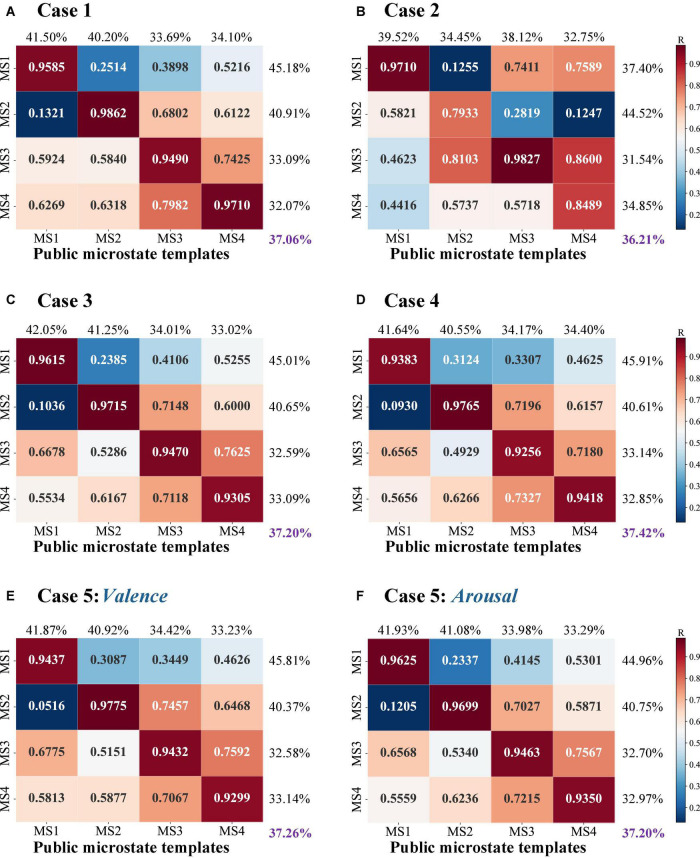
The spatial correlation results with the public microstate templates. The heatmap is a visual display of the calculated correlation coefficient of topographical similarity. In a range from 0 to 1, a higher correlation coefficient is marked as red indicating a close topographical similarity between identified microstates and the public microstate templates, whereas a low correlation coefficient is marked as blue referring to a topographical dissimilarity. Here, **(A–D)** refer to the results of the identified EEG microstate templates by Case 1, 2, 3, and 4; **(E)** and **(F)** refer to the results of the identified valence-based and arousal-based EEG microstate templates by Case 5.

To identify the attribution of each identified EEG microstate to the canonical templates, the spatial correlations with the standard (public) microstate templates are measured. It is a quantitive evaluation of microstate quality in terms of topographical configuration. The observed task-state microstates in Milz et al. (2016) were adopted here as a standard template for microstate quality evaluation. This set of microstate templates was identified from 61 subjects’ EEG data during three thinking tasks and is available online.^1^ For each topographical clustering strategy, the spatial correlation results between identified EEG microstates and standard templates are presented in a 4×4 correlation coefficient matrix (as shown in Figure 4). A high correlation with the standard templates indicates an optimal set of microstates could be obtained with maximized between-cluster and minimized within-cluster relationships. Based on the 4×4 correlation coefficient matrix, both individual microstate performance of each microstate and overall microstate performance across all microstates are evaluated for spatial correlation calculation. For individual microstate performance evaluation, the ratio of each diagonal value to the corresponding sum of correlation coefficients located in the same row/column is calculated for template comparison. For overall microstate performance evaluation, the ratio of the sum of the diagonal values to the total of correlation coefficients is calculated for case comparison. Larger ratio values reveal better microstate quality with closer topographical similarity to the standards. Take Figure 4A as an example. In the evaluation of the individual microstate performance, the ratios of MS1, MS2, MS3, and MS4 are calculated, respectively. For MS1, the ratio of 0.9585 to the sum of correlation coefficients located in the first row is 45.18%, while the ratio of 0.9585 to the sum of correlation coefficients located in the first column is 41.50%. For MS2, 40.91 and 40.20% are the ratios of 0.9862 to the sum of correlation coefficients located in the second row and column, respectively. For MS3, the corresponding calculated ratios are 33.09 and 33.69%. For MS4, the ratios of 0.9710 to the sum of correlation coefficients located in the fourth row and column are 32.07 and 34.10%, respectively. In the evaluation of the overall microstate performance, the ratio of a sum of 0.9585, 0.9862, 0.9490, and 0.9710 to the sum of all the correlation coefficients in the 4×4 matrix is equal to 37.06%. Similarly, the overall microstate performance evaluations are conducted on the other cases. In all, the corresponding overall microstate performance evaluation results of Case1, Case2, Case3, and Case4 are 37.06, 36.21, 37.20, and 37.42%, respectively. For Case 5, the spatial correlation ratio of the valence-based microstates is 37.26% and that of arousal-based microstates is 37.42%. Among the results, Case 4 and Case 5’s results show the 45 highest correlation relationships with the standard microstate templates. The lowest spatial correlation is Case 2 (36.21%), especially for the MS2 (*R* = 0.7933) and MS4 (*R* = 0.8489).

**FIGURE 5:**
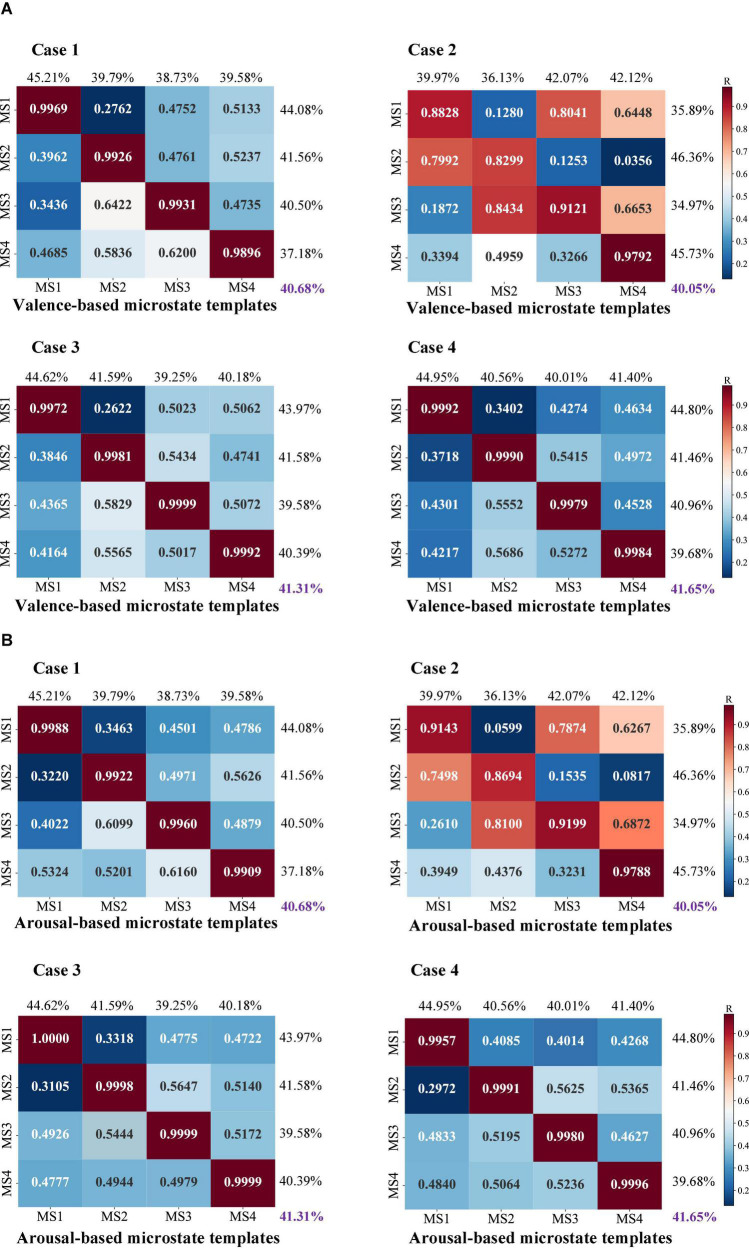
The spatial correlation results between four bottom-up topographical clustering strategies and task-driven-based top-down clustering for **(A)** valence-based and **(B)** arousal-based microstate templates of Case 5. The heatmap is a visual display of the calculated correlation coefficient of topographical similarity between identified microstates and emotion-related microstate templates. In a range from 0 to 1, a higher correlation coefficient is marked as red indicating a close topographical similarity, whereas a low correlation coefficient is marked as blue indicating a low similarity.

The EEG microstate analysis on task-state EEG recordings should also be able to reflect the mental state changes related to the task. To check the involved task information in the microstates and investigate whether the task-free clustering results share similar neuronal activity patterns with the task-guided one, we also calculate the spatial correlations of the detected microstates by the bottom-top-based data-driven approaches (Case 1, Case 2, Case 3, and Case 4) with the detected microstates by the top-down-based task information-guided approach (Case 5). Similarly, the ratio of the sums of diagonal values to the total of correlation coefficients is calculated as an overall estimation. In consideration of the spatial correlation calculation with valence-based microstates (Figure 5A), a high correlation is observed in Case 1, Case 3, and Case 4 with a mean ratio of 40.68, 41.31, and 41.65%. For the spatial correlation calculation with arousal-based templates (Figure 5B), we find that the microstates of Case 3 (41.26%) and Case 4 (41.57%) achieve a high microstate quality in potential configuration. On the other hand, Case 2 fails to achieve ideal results in microstate quality measurement. Compared to the other cases, the corresponding within-cluster and between-cluster distances are relatively poor (valence-based: 40.05%; arousal-based: 40.67%).

Overall, the evaluation results in terms of microstate quality show the detected microstates using Case 3 and Case 4 outperform the other topographical strategies, where a high spatial correlation relationship with the existing standard templates is observed and a similar topography configuration with the task-based templates is also found.

#### Global Explained Variance

The performance of fitting the identified microstate templates back into EEG data will significantly influence the results of EEG microstate segmentation. GEV measures how much percentage of original EEG data can be represented by the given set of EEG microstate templates. It is commonly used to reflect the topographical quality in EEG microstate segmentation. Table 1A shows the calculated GEV values based on the detected microstates using five different microstate detection methods with different topographical clustering strategies. The results show the calculated GEV values are mainly located in the range from 0.6 to 0.7, and the Cronbach’s α values across 5 topographical clustering strategies are larger than 0.9 (0.9817 0.0040). No significant difference is observed in the calculated GEV values when different topographical clustering strategies are adopted. Besides, when we calculate the GEV proportions of each microstate across all EEG microstate time series (Table 1B), we find that in most cases MS3 and MS4 occupy the highest GEV proportions (the total proportion is about 60%). For MS1 and MS2, the corresponding GEV proportions over the EEG microstate time series are relatively low, where each of them is around 20%. However, a different pattern is observed in Case 2, where MS1’s GEV is larger than MS4’s. The GEV calculation results show MS1 plays a more dominant role in representing EEG into EEG microstate time series compared to MS4. In the GEV proportion calculation, a larger proportion is observed on MS4, instead of MS1. The inconsistent results between the GEV values and GEV proportions suggest that the microstate quality of the detected microstates in Case 2 is not good enough.

**TABLE 1:** The GEV results on different microstate detection methods with different topographical clustering strategies.

(A) GEV values (mean ± standard deviation)						

	**Case 1**	**Case 2**	**Case 3**	**Case 4**	**Case 5 *valence***	**Case 5 *arousal***
MS1	0.0780 ± 0.0382	0.1209 ± 0.0424	0.0758 ± 0.0375	0.0721 ± 0.0369	0.0712 ± 0.0369	0.0763 ± 0.0375
MS2	0.0839 ± 0.0318	0.0593 ± 0.0309	0.0919 ± 0.0348	0.0919 ± 0.0346	0.0984 ± 0.0362	0.0893 ± 0.0342
MS3	0.1635 ± 0.0551	0.2084 ± 0.0706	0.1752 ± 0.0587	0.1665 ± 0.0554	0.1742 ± 0.0578	0.1738 ± 0.0584
MS4	0.1780 ± 0.0562	0.1176 ± 0.0476	0.1617 ± 0.0535	0.1726 ± 0.0552	0.1608 ± 0.0541	0.1652 ± 0.0539
Total	0.6711 ± 0.0497	0.6738 ± 0.0523	0.6733 ± 0.0512	0.6725 ± 0.0508	0.6732 ± 0.0511	0.6732 ± 0.0511

**(B) GEV proportions.**					

**Proportion (100%)**		**MS1**	**MS2**	**MS3**	**MS4**

Task-Free		Case 1	18.91%	18.93%	30.62%	31.55%
		Case 2	23.47%	16.15%	33.86%	26.52%
		Case 3	18.05%	20.16%	30.79%	31.00%
		Case 4	17.67%	19.67%	30.59%	32.07%
Task-Guided		Case 5 (*valence)*	17.51%	20.80%	30.91%	30.78%
		Case 5 *(arousal)*	18.11%	19.76%	30.76%	31.37%
	Cronbach’s α		0.9784	0.9782	0.9840	0.9862

### Task Efficacy

Through the statistical analysis between low and high emotional groups, we evaluate the task efficacy of each of the microstate features detected in each of the presented microstate detection methods. It is a reflection of dynamic changes in microstate patterns during tasks. As valence and arousal are two independent emotion dimensions, the task efficacy is separately examined on these two dimensions.

Inspecting the microstate activity differences in the valence dimension, as shown in Table 2, the task-driven-based top-down topographical clustering (Case 5) shows that MS1 coverage (*p* = 0.0477), MS1 duration (*p* = 0.0060), and MS2 duration (*p* = 0.0118) of the high valence group are higher than that of the low valence group. But MS4 coverage (*p* = 0.0043) and occurrence (*p* = 0.0003) of the high valence group are significantly lower than the low valence group. We also explore the feature patterns in the trial-subject-sequence-based (Case 1), the subject-trial-sequence-based (Case 2), the single-trial-based subject-trial-sequence-based (Case 3), and the random-grouping-based (Case 4) bottom-up topographical clustering methods. For Case 1, we only observe one consistent microstate pattern that a larger MS4 occurrence (*p* = 0.0379) is found in the high valence group. For Case 2, MS1 coverage (*p* = 0.0411) and duration (*p* = 0.0104) of the high valence group are larger but the MS4 coverage (*p* = 0.0007), duration (*p* = 0.0144), and occurrence (*p* = 0.0001) are lower than the low valence group. For Case 3, a more similar pattern to Case 5 in valence-related microstate activities is observed. There is higher MS1 coverage (*p* = 0.0420), MS1 duration (*p* = 0.0057), and MS2 duration (*p* = 0.0092), but lower MS4 coverage (*p* = 0.0042) and occurrence (*p* = 0.0005), in the high valence group comparing to the low-level group. Similarly, based on the microstate templates identified in Case 4, the statistical differences in microstate activities between low- and high-level valence states show that high valence group corresponds to larger MS1 duration (*p* = 0.0092) and MS2 duration (*p* = 0.0092) but lower MS4 coverage (*p* = 0.0092) and occurrence (*p* = 0.0092).

**TABLE 2:** The task efficacy results in valence dimension (*p < 0.05, **p < 0.01, FDR).

	(A) Coverage			
	MS1	MS2	MS3	MS4
**Case 1**				
Low valence	0.1875 ± 0.0631	0.1877 ± 0.0491	0.3056 ± 0.0665	0.3192 ± 0.0750
High valence	0.1907 ± 0.0583	0.1910 ± 0.0483	0.3069 ± 0.0660	0.3115 ± 0.0715
Statistics	–1.2999	–1.4162	–0.6379	1.8083
**Case 2**				
Low valence	0.2319 ± 0.0519	0.1596 ± 0.0535	0.3366 ± 0.0731	0.2719 ± 0.0802
High valence	0.2377 ± 0.0519	0.1635 ± 0.0554	0.3408 ± 0.0740	0.2580 ± 0.0772
Statistics	–2.0423	–1.3105	–1.1146	3.3921*
**Case 3**				
Low valence	0.1777 ± 0.0595	0.1997 ± 0.0520	0.3066 ± 0.0634	0.3160 ± 0.0777
High valence	0.1835 ± 0.0598	0.2037 ± 0.0519	0.3093 ± 0.0689	0.3036 ± 0.0739
Statistics	–2.0339	–1.1233	–0.4174	2.8621*
**Case 4**				
Low valence	0.1742 ± 0.0598	0.1949 ± 0.0508	0.3045 ± 0.0624	0.3264 ± 0.0762
High valence	0.1794 ± 0.0600	0.1985 ± 0.0504	0.3075 ± 0.0674	0.3146 ± 0.0725
Statistics	–1.8258	–0.9618	–0.5821	2.6645*
**Case 5**				
Low valence	0.1724 ± 0.0600	0.2062 ± 0.0525	0.3076 ± 0.0626	0.3137 ± 0.0778
High valence	0.1778 ± 0.0601	0.2100 ± 0.0518	0.3107 ± 0.0685	0.3015 ± 0.0745
Statistics	–1.9796	–1.0269	–0.5620	2.8555*

	**(B) Duration**			
	**MS1**	**MS2**	**MS3**	**MS4**

**Case 1**				
Low valence	64.6320 ± 7.6889	64.4197 ± 5.9984	79.5613 ± 11.2111	80.6605 ± 11.9049
High valence	65.1246 ± 6.9375	64.6901 ± 5.8365	79.7905 ± 11.2312	79.6766 ± 11.0350
Statistics	–1.8721	–1.0825	–0.6482	1.2493
**Case 2**				
Low valence	69.1943 ± 6.6213	61.6431 ± 6.2360	82.9398 ± 12.5854	74.3199 ± 11.2099
High valence	70.3438 ± 7.2465	62.2864 ± 6.5304	84.2994 ± 13.3318	73.0133 ± 10.8779
Statistics	–2.5624*	–1.8436	–1.9423	2.4473*
**Case 3**				
Low valence	63.4815 ± 7.0181	65.5455 ± 6.5484	79.2349 ± 10.4002	80.0604 ± 12.3229
High valence	64.5303 ± 7.3439	66.3463 ± 6.5271	80.4348 ± 11.7304	78.7423 ± 11.2168
Statistics	–2.7661**	–2.6061**	–1.3383	1.8643
**Case 4**				
Low valence	63.1787 ± 7.0845	64.9402 ± 6.4114	78.9501 ± 10.4466	81.3303 ± 12.3963
High valence	64.1381 ± 7.3568	65.7845 ± 6.2856	80.1698 ± 11.6227	80.1675 ± 10.9907
Statistics	–2.6225*	–2.7478*	–1.4367	1.2166
**Case 5**				
Low valence	62.9790 ± 7.0504	66.1826 ± 6.5955	79.2912 ± 10.3204	79.7048 ± 12.4028
High valence	64.0441 ± 7.4066	67.0386 ± 6.5729	80.4988 ± 11.6674	78.5148 ± 11.3720
Statistics	–2.7475*	–2.5192	–1.3623	1.6941

	**(C) Occurrence**			
	**MS1**	**MS2**	**MS3**	**MS4**

**Case 1**				
Low valence	2.8398 ± 0.6814	2.8780 ± 0.5913	3.8085 ± 0.4384	3.9187 ± 0.4391
High valence	2.8759 ± 0.6383	2.9184 ± 0.5809	3.8135 ± 0.4479	3.8722 ± 0.4307
Statistics	–0.9220	–1.5460	–0.5326	2.0763*
**Case 2**				
Low valence	3.3208 ± 0.5343	2.5390 ± 0.6505	4.0256 ± 0.4335	3.5964 ± 0.5686
High valence	3.3509 ± 0.5106	2.5707 ± 0.6591	4.0125 ± 0.4194	3.4742 ± 0.5528
Statistics	–1.0644	–1.0842	0.6471	3.8958**
**Case 3**				
Low valence	2.7421 ± 0.6663	3.0066 ± 0.6069	3.8393 ± 0.4385	3.9049 ± 0.4522
High valence	2.7876 ± 0.6568	3.0312 ± 0.5852	3.8116 ± 0.4448	3.8140 ± 0.4646
Statistics	–1.4067	–0.5572	1.5072	3.4524*
**Case 4**				
Low valence	2.6979 ± 0.6702	2.9624 ± 0.6015	3.8282 ± 0.4325	3.9760 ± 0.4285
High valence	2.7388 ± 0.6620	2.9812 ± 0.5804	3.8034 ± 0.4378	3.8872 ± 0.4499
Statistics	–1.0972	–0.2368	1.3515	3.5872**
**Case 5**				
Low valence	2.6791 ± 0.6782	3.0755 ± 0.5952	3.8504 ± 0.4372	3.8936 ± 0.4485
High valence	2.7180 ± 0.6655	3.0957 ± 0.5736	3.8270 ± 0.4465	3.7983 ± 0.4662
Statistics	–1.0349	–0.3405	1.3069	3.5917**

For the arousal dimension, as shown in Table 3, the task-driven-based top-down topographical clustering (Case 5) shows that MS3 coverage (*p* = 0.0317) and duration (*p* = 0.0108) of the high arousal group are higher than the low arousal group. While MS4 coverage (*p* = 0.0034), duration (*p* = 0.0073), and occurrence (*p* = 0.0044) are significantly lower when comparing the high arousal group to the low-level one. Comparison studies on the feature patterns are also conducted on the bottom-up topographical clusterings (Case 1–4). For Case 1, we only observe the arousal-related statistical differences on MS3, where MS3 coverage (*p* = 0.0147) and occurrence (*p* = 0.0203) are larger in the high arousal group. No significant difference is observed in MS4. For Case 2, the high arousal group yields to larger MS3 coverage (*p* = 0.0335) and duration (*p* = 0.0209), but lower MS4 coverage (*p* = 0.0002), duration (*p* = 0.0003), and occurrence (*p* = 0.0004) than the low arousal group. A similar activity pattern is found in Case 3 that the high arousal group corresponds to higher MS3 coverage (*p* = 0.0003), duration (*p* = 0.0003), and lower MS4 coverage (*p* = 0.0003), duration (*p* = 0.0003), and occurrence (*p* = 0.0003). However, in Case 4, the microstate activity difference between low and high arousal groups is only presented in MS4, where MS4 coverage (*p* = 0.0106) and occurrence (*p* = 0.0056) of the high arousal group are higher than the low arousal group.

**TABLE 3:** The task efficacy results in arousal dimension (*p < 0.05, **p < 0.01, FDR).

	(A) Coverage			
	MS1	MS2	MS3	MS4
**Case 1**				
Low arousal	0.1895 ± 0.0619	0.1894 ± 0.0498	0.3023 ± 0.0678	0.3188 ± 0.0757
High arousal	0.1886 ± 0.0598	0.1892 ± 0.0477	0.3100 ± 0.0645	0.3122 ± 0.0710
Statistics	0.2563	0.6788	–2.4391*	1.2217
**Case 2**				
Low arousal	0.2329 ± 0.0511	0.1604 ± 0.0548	0.3342 ± 0.0755	0.2725 ± 0.0799
High arousal	0.2366 ± 0.0527	0.1625 ± 0.0540	0.3430 ± 0.0713	0.2580 ± 0.0775
Statistics	–1.9196	–0.7224	–2.1283	3.6303**
**Case 3**				
Low arousal	0.1795 ± 0.0599	0.1996 ± 0.0534	0.3047 ± 0.0677	0.3162 ± 0.0778
High arousal	0.1815 ± 0.0595	0.2036 ± 0.0505	0.3111 ± 0.0645	0.3038 ± 0.0739
Statistics	–0.5757	–1.1763	–2.2006	2.9272*
**Case 4**				
Low arousal	0.1755 ± 0.0602	0.1948 ± 0.0522	0.3034 ± 0.0663	0.3263 ± 0.0765
High arousal	0.1779 ± 0.0596	0.1985 ± 0.0491	0.3085 ± 0.0634	0.3151 ± 0.0724
Statistics	–0.6806	–1.1705	–1.8744	2.5562
**Case 5**				
Low arousal	0.1800 ± 0.0595	0.1955 ± 0.0530	0.3044 ± 0.0679	0.3200 ± 0.0775
High arousal	0.1821 ± 0.0593	0.1996 ± 0.0502	0.3107 ± 0.0647	0.3075 ± 0.0734
Statistics	–0.5993	–1.2188	–2.1479	2.9262*

	**(B) Duration**			
	**MS1**	**MS2**	**MS3**	**MS4**

**Case 1**				
Low arousal	64.9348 ± 7.6470	64.5584 ± 6.0885	79.1522 ± 11.3010	80.8930 ± 12.2272
High arousal	64.8078 ± 7.0182	64.5435 ± 5.7522	80.1879 ± 11.1180	79.4800 ± 10.6881
Statistics	0.0864	0.0449	–1.8807	1.6788
**Case 2**				
Low arousal	69.4012 ± 6.9642	61.8284 ± 6.3813	82.9017 ± 12.9593	74.5860 ± 11.3363
High arousal	70.0995 ± 6.9293	62.0808 ± 6.3934	84.2906 ± 12.9450	72.7944 ± 10.7242
Statistics	–1.9539	–0.2640	–2.3099	3.5508**
**Case 3**				
Low arousal	63.8640 ± 7.2666	65.7942 ± 6.6146	79.2145 ± 11.2765	80.2174 ± 12.2810
High arousal	64.1155 ± 7.1255	66.0725 ± 6.4830	80.4139 ± 10.8525	78.6318 ± 11.2845
Statistics	–0.5428	–0.8298	–2.6936*	2.3969
**Case 4**				
Low arousal	63.4961 ± 7.2292	65.1642 ± 6.3683	79.1083 ± 11.1606	81.5000 ± 12.2706
High arousal	63.7908 ± 7.2354	65.5337 ± 6.3558	79.9714 ± 10.9218	80.0392 ± 11.1626
Statistics	–0.5978	–1.2586	–1.9035	1.9494
**Case 5**				
Low arousal	63.9495 ± 7.2931	65.4048 ± 6.4612	79.1960 ± 11.3402	80.8319 ± 12.3519
High arousal	64.1662 ± 7.1453	65.6350 ± 6.4818	80.3416 ± 10.9173	79.0640 ± 11.1837
Statistics	–0.4958	–0.6904	–2.5481*	2.6841*

	**(C) Occurrence**			
	**MS1**	**MS2**	**MS3**	**MS4**

**Case 1**				
Low arousal	2.8582 ± 0.6666	2.8966 ± 0.6001	3.7841 ± 0.4553	3.9015 ± 0.4221
High arousal	2.8564 ± 0.6556	2.8986 ± 0.5729	3.8375 ± 0.4289	3.8909 ± 0.4487
Statistics	0.0512	0.7167	–2.3208*	0.3278
**Case 2**				
Low arousal	3.3252 ± 0.5136	2.5408 ± 0.6652	3.9959 ± 0.4447	3.5924 ± 0.5548
High arousal	3.3454 ± 0.5323	2.5678 ± 0.6442	4.0423 ± 0.4068	3.4823 ± 0.5683
Statistics	–0.8359	–0.7925	–1.5642	3.5065**
**Case 3**				
Low arousal	2.7519 ± 0.6667	2.9912 ± 0.6174	3.8115 ± 0.4493	3.9004 ± 0.4495
High arousal	2.7763 ± 0.6573	3.0456 ± 0.5740	3.8401 ± 0.4337	3.8216 ± 0.4679
Statistics	–0.6593	–1.3315	–1.1814	3.0548*
**Case 4**				
Low arousal	2.7026 ± 0.6734	2.9488 ± 0.6155	3.8024 ± 0.4426	3.9663 ± 0.4346
High arousal	2.7328 ± 0.6593	2.9939 ± 0.5657	3.8297 ± 0.4274	3.8998 ± 0.4453
Statistics	–0.8093	–1.1076	–1.1751	2.7681*
**Case 5**				
Low arousal	2.7568 ± 0.6613	2.9480 ± 0.6206	3.8090 ± 0.4513	3.9190 ± 0.4416
High arousal	2.7847 ± 0.6527	3.0044 ± 0.5754	3.8383 ± 0.4365	3.8492 ± 0.4606
Statistics	–0.7605	–1.4606	–1.3268	2.8475*

**FIGURE 6:**
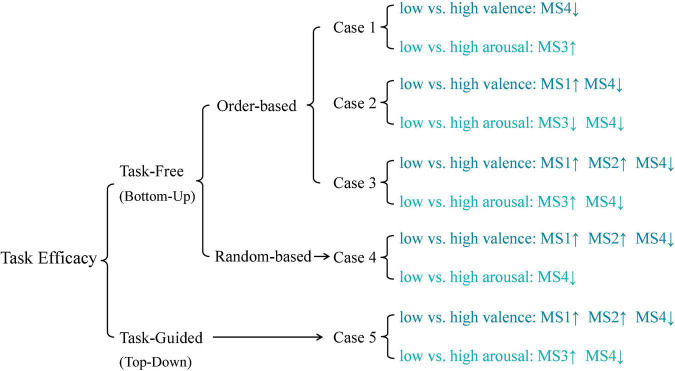
A summary of task efficacy results.

A summary of the task efficacy results across different types of topographical clustering strategies is reported in Figure 6. Compared to the statistical results of Case 5, it is found that the microstate detection results of Case 3 perform the most similar patterns in the reflection of emotion changes for both valence and arousal dimensions. On the other hand, the common feature patterns between Case 1 and Case 5 are the least.

### Computational Efficiency

In microstate analysis, EEG microstate detection is a very time-consuming part. It is also very important to check the computational efficiency of different topographical clustering strategies. To make sure the measured time costs are comparable across different microstate detection methods, we use the same predefined parameters in all the different topographical clustering strategies and compare all the results on the same database (total 1280 trials). In the first-step clustering, a modified k-means algorithm with the cluster number *c* ranging from 2 to 8 and the iteration number *I* of 1000 is used. For the second-step clustering, another modified k-means clustering is conducted with the cluster number *c* ranging from 2 to 8 and the iteration number *I* of 5000. The computed computational efficiency results of different microstate detection methods with different topographical clustering strategies are presented in Table 4. It is found Case 3 is the most time-consuming method, taking a total of 607,572 s (approximately 168 h). Similarly, the total cost time in Case 5 in terms of different emotional dimensions (valence and arousal) are also 605,476 and 605,531 s, respectively. Both Case 3 and Case 5 are based on trial-based topographical clustering strategy, thus the computational efficiency is lower than the other non-trial-based topographical clustering strategies. For Case 1 and Case 4, the corresponding computational efficiency results are 224,928 and 211,592 s, respectively. Case 2 has the highest computational efficiency, with a total cost of 191,233 s. The time cost difference between Case 1/4 and Case2 is mostly determined by the calculation amount in the first-step clustering. All the reported results are calculated on the same computer (Intel^®^ Core™ i7-10700 CPU@2.90 GHz, 32 GB RAM).

**TABLE 4:** The computational efficiency results of different microstate detection methods with different topographical clustering strategies.

		First-step clustering	Second-step clustering	Total
Task-free	Case 1	224,896 s	32 s	224,928 s
	Case 2	191,200 s	33 s	191,233 s
	Case 3	604,160 s	3,412 s	607,572 s
	Case 4	211,560 s	32 s	211,592 s
Task-Guided	Case 5 (*valence)*	604,160 s	682 s (low valence)	605,476 s
			634 s (high valence)	
	Case 5 *(arousal)*	604,160 s	679 s (low arousal)	605,531 s
			692 s (high arousal)	

*c, the number of clusters; I, the number of iteration. Here, c ranges from 2 to 8, and I is set to 1000.*

## Discussion and Conclusion

In this study, we present four data-driven bottom-up-based and 1 prior-knowledge-guided top-down based topographical clustering strategies for naturalistic task-state EEG microstate detection. The performance discrepancies among different bottom-up-based strategies and between bottom-up and top-down manners are quantitatively examined from the perspectives of microstate quality, task efficacy, and computational efficiency. Therefore, the effect of different EEG data grouping order, clustering arrangement, and the use of task-guided information on microstate detection performance is mainly discussed. By examining these available clustering strategies, we tend to explore an optimal bottom-up based topographical clustering strategy for task-state EEG analysis to obtain similar performance in detecting neuronal activity dynamics using the top-down based clustering strategy.

The results show that the task-driven-based top-down topographical clustering strategy performs well in reflection of emotion-related neural changes. The corresponding identified microstate templates share high similarities in the topography distribution property with the existing findings. It was found that MS1 and MS2 are functionally associated with visual and verbal processing, and MS3 and MS4 are related to the functional activity of default mode and dorsal attention networks corresponding to the high-level brain activity of goal-directed perception processing (Milz et al., 2016; Michel and Koenig, 2018). Case 5’s results also reflect such a comprehensive representation difference in dynamic brain activities, where higher MS1, MS2, and lower MS4 activities are observed in the high valence group, while higher MS3 and lower MS4 activities are found in the high arousal group. These results are consistent with the patterns of brain activities under different valence and arousal states in the previous studies. For example, Nummenmaa et al. (2012) adopted a naturalistic video-based paradigm for emotion induction and worked on the relationship between the activation state of large-scale brain networks and self-assessment of evoked emotional states. Their results presented an intersubjective synchronization in brain activation patterns under the emotional dimensions of valence and arousal. A close correlation was found between a low valence state and an increased activity of the default mode network, and between a high arousal state and increased activity of the dorsal attention network. Given the observations between EEG microstates and functional brain networks, the obtained results support that the top-down approach with prior-knowledge guidance performs superior in the task-state EEG microstate analysis.

When we compare the bottom-up approaches without prior-knowledge guidance to Case 5’s results (Table 5), it is found that the single-trial-based bottom-up topographical clustering (Case 3) achieves the most similar results with high microstate quality and task efficacy. But the corresponding computational process of Case 3 is quite time-consuming. Therefore, Case 3 is recommended for bottom-up microstate clustering when the prior knowledge is not available and the computational efficiency is not highly demanded. On the other hand, the random-grouping-based bottom-up topographical clustering (Case 4) also shares a comparable performance in task-state EEG microstate analysis. For the applications with a high requirement of computational efficiency, Case 4 is suggested for reliable microstate detection.

**TABLE 5:** An overview of the performance comparison among different bottom-up clustering strategies.

	Microstate quality	Task efficacy	Computational efficiency
Case 1	Average	Average	Average
Case 2	Poor	Poor	Excellent
Case 3	Excellent	Excellent	Poor
Case 4	Excellent	Good	Good

For the commonly used trial-subject-sequence-based bottom-up topographical clustering (Case 1) in rest-state EEG microstate analysis, the corresponding performance on task-state EEG microstate analysis fails to achieve promising results as in Case 3 and Case 4. These results show the necessity of exploring suitable topographical clustering strategies for task-state EEG analysis, instead of directly coping with the same approach used in rest-state EEG analysis. It inspires us to explore more flexible and well-designed topographical clustering strategies in microstate detection for task-state EEG recordings, which could better address the complexity and variability issues in task manipulation. Also, we believe a well-performed microstate detection method would also work well on the rest-state EEG microstate analysis.

In this current work, we mainly focus on the effect of topographical clustering strategies on microstate detection performance. When comparing our results with the current task-state EEG microstate analysis, we found that EEG microstate analysis is powerful in characterizing dynamic neural response patterns that effected by the naturalistic tasks. On the other hand, Han et al. (2020) conducted a task-state EEG microstate analysis in a picture-based event-related potential (ERP) facial attractiveness judgment experiment and observed a total of six EEG microstates from the task-state ERP data that is different from our observations. There would be two possible reasons: (1) Different experimental paradigms. In our work, we focus on task-state EEG signals during naturalistic tasks, using various audio-visual materials to elicit different emotions. Different tasks would lead to a different pattern of neural activities (Milz et al., 2016) and further lead to different microstate results. (2) Different EEG processing methods. Han et al.’s (2020) work was carried out on the ERP data, where the polarity of potential topographies was considered. Our work is based on the raw recording of spontaneous EEG data for studying the naturalistic and dynamic brain changes under different emotional states. Here, the potential polarity is ignored as EEG topographies with inverted polarity would share a synchronized activation pattern of neuronal ensembles (Lehmann and Koenig, 1997; Mishra et al., 2020). Additionally, Khanna et al. (2014) have validated a high consistency between the microstate topographies identified from different numbers of electrode arrays. Their results observed that the microstate features extracted from 8 and 19 electrodes shared a high test-retest reliability with that from 30 electrodes. As an extension of our current work, the integration analysis on different combinations of clustering strategies and electrode sets could be considered to further examine the performance efficiency and stability of identified task-state microstates for brain dynamics study in naturalistic tasks.

In all, our work evaluates the effects of microstate detection across different topographical clustering strategies on EEG microstate analysis under a naturalistic paradigm. We present four data-driven bottom-up topographical clustering strategies and one task-driven-based top-down topographical clustering strategy, compare the performance under different cluster analytical processes, and discuss the performance impact when prior knowledge about the task information is utilized in the microstate detection. Besides, we present a systematic performance evaluation protocol to quantify the identified microstates from three different perspectives (microstate quality, task efficacy, and computational efficiency). The results show that task-driven-based top-down topographical clustering outperforms the data-driven bottom-up topographical clustering strategies. Among the data-driven bottom-up topographical clustering strategies, a single-trial-based bottom-up approach (Case 3) performs the best in terms of both microstate quality and task efficacy, but the computational efficiency is relatively low. This work presents a systematic study to analyze these available topographical clustering strategies and suggests a good microstate detection for task-state EEG microstate analysis.

## Data Availability Statement

The datasets presented in this study can be found in online repositories. The names of the repository/repositories and accession number(s) can be found below: http://www.eecs.qmul.ac.uk/mmv/datasets/deap/.

## Ethics Statement

All the human data used in the manuscript are from a published public database (DEAP). The patients/participants provided their written informed consent to participate in this study.

## Author Contributions

WH, ZZ, and ZL conceived and designed the original research. WH was responsible for data processing and data analysis. All authors contributed to writing and editing the manuscript, contributed to the article, and approved the submitted version.

## Conflict of Interest

The authors declare that the research was conducted in the absence of any commercial or financial relationships that could be construed as a potential conflict of interest.

## Publisher’s Note

All claims expressed in this article are solely those of the authors and do not necessarily represent those of their affiliated organizations, or those of the publisher, the editors and the reviewers. Any product that may be evaluated in this article, or claim that may be made by its manufacturer, is not guaranteed or endorsed by the publisher.

## Footnotes

1
https://github.com/keyinst/keypy
